# Social Network Analysis and Churn Prediction in Telecommunications Using Graph Theory

**DOI:** 10.3390/e22070753

**Published:** 2020-07-09

**Authors:** Stefan M. Kostić, Mirjana I. Simić, Miroljub V. Kostić

**Affiliations:** 1 School of Electrical Engineering, University of Belgrade, 11000 Belgrade, Serbia; mira@etf.rs; 2 Statistical Office of the Republic of Serbia, 11000 Belgrade, Serbia; miroljub.kostic@stat.gov.rs

**Keywords:** machine learning, data mining, call data record, churn prediction, graph theory, social network analysis

## Abstract

Due to telecommunications market saturation, it is very important for telco operators to always have fresh insights into their customer’s dynamics. In that regard, social network analytics and its application with graph theory can be very useful. In this paper we analyze a social network that is represented by a large telco network graph and perform clustering of its nodes by studying a broad set of metrics, e.g., node in/out degree, first and second order influence, eigenvector, authority and hub values. This paper demonstrates that it is possible to identify some important nodes in our social network (graph) that are vital regarding churn prediction. We show that if such a node leaves a monitored telco operator, customers that frequently interact with that specific node will be more prone to leave the monitored telco operator network as well; thus, by analyzing existing churn and previous call patterns, we proactively predict new customers that will probably churn. The churn prediction results are quantified by using top decile lift metrics. The proposed method is general enough to be readily adopted in any field where homophilic or friendship connections can be assumed as a potential churn driver.

## 1. Introduction

Data mining represents the process of extracting knowledge hidden in large volumes of data and identifying potentially useful and understandable data. Telecommunications industry generates enormous amounts of data that are suitable for data mining. Applying data mining in telecommunications can improve fraud detection and lower churn rates [1,2]. Churn is a measure of customer attrition; it refers to the proportion of subscribers who leave an operator during a given period. Initially, growth in telecommunications market was exponential and churn was not regarded as a problem, but nowadays telco market is mature and increased competition leads telco companies to focus on keeping their existing customers. Telecommunications market became even more competitive with the new number portability feature introduction.

Social network analytics [3,4] set out one of the possible applications of data mining in telecommunications. A social network is a set of nodes (that may represent people, organizations or other social entities), that are connected by a set of relationships, such as friendship, affiliation or information exchange [5]. As a topic, social network analytics has been studied for years, and there can be found a significant amount of papers on mining real world graphs (e.g., Internet, SMS, and voice graphs) that are formed by interactions amongst individuals of a social network [6]. There are several important papers [7,8,9] on social network analysis in telecommunications. All of the mentioned papers are based on the fact that graphs induced by people calling each other can allow telco operators to better understand interaction behavior of their customers. Since it has been calculated that it is much cheaper to retain an existing customer compared to the cost of acquiring a new one, one of the main tasks for a successful telco provider is to control its churn rate [10]. Several methods were proposed in [11,12], but we will present a new method that combines the methods of social network graph analysis [13] and customer profiling and clustering [14].

In this paper, we will present a new reactive method for churn prediction in the telecommunications market that is based on the social network analysis principle. We will demonstrate that homophilic rules can affect churn in telco operator networks due to the fact that friends of churners are more likely to churn themselves and, additionally, we will quantify this effect.

By analyzing Call Data Records (CDRs), we will calculate nine metrics that will describe different client behavior. Those nine node weights will be used to create a total of five customer clusters: Follower, Standard, Leader, Core and Important customer clusters. We will demonstrate that neighbor churn probability is not the same in case when clients from different clusters churn. Precisely, customers that interact with Leader and Important nodes (i.e., influencers) are more prone to leave our monitored network (i.e., to perform churn) in case when an influencer leaves the network.

The contributions of this paper can be summarized in the way as follows:

- We study a broad set of metrics to reveal various important telco network call graph structural properties;
- We define three sets of metrics that can precisely describe each element of the network call graph—directed and undirected metrics, as well as articulation point metrics; we will demonstrate that, even though telco networks are directed by nature, the undirected metrics as well can provide us with a meaningful and important additional insight regarding churn prediction;
- We introduce a new method based on graph theory metrics for detection of not valid (non-human) nodes of the network call graph;
- We present a model based on real-life data that can provide important business insights and help design better strategies for struggle against churn for mobile telco operators;
- We define a method for combining two separate clustering models results into a unified scale that presents a final model;
- Due to the limited scope of base variables analyzed in our paper (only number and duration of calls, as well as information regarding influencer churn), it is possible to combine our method with other state-of-art models that focus on other metrics (e.g., some customer satisfaction based churn model), so that the churn prediction success rate could be increased even more.

The paper is further organized as follows. The next section presents the comparison with the related work. Section 3 contains a brief description of the idea behind the entire process. Additionally, in Section 3 we also present metrics that will be used for numerical description of the call graph nodes. In Section 4 we describe our datasets, the process of creation of the call graphs and a method for eliminating not valid nodes. The process of Mobile Station International Subscriber Directory Number (MSISDN—a number uniquely identifying a subscription in a mobile network) clustering is also presented in Section 4. In Section 5, we define the final prediction model, validate it by using real-life churn data and present the churn prediction model results. In Section 6, the comparison of our proposed modelling method with other modelling methods is presented. Finally, we conclude the paper with Section 7.

## 2. Related Work

The availability of mobile phone communications has provided researchers with numerous options to analyze mobile networks. Churn prediction is one of the most important tasks for any modern telecommunications company. One approach that exploits important benefits of modelling the interactions between customers is presented in [15]. The system presented in [15] is able to overcome the limitation of social-network based approaches that require knowing which customers have churned recently. The achieved ten decile lift presented in [15] is equal to 2.5.

In article [12], the authors investigated several issues related to the combination of unsupervised clustering techniques and decision trees with boosting. They studied five clustering techniques for hybridization and evaluated the models in terms of top decile lift. The maximum achieved lift is approximately 2.6.

A simple, yet effective, diffusion based approach that exploits social relationships to identify a significant fraction of churners in the network is presented in [11]. Here influences are purely derived from the call volumes between individuals and the achieved lift is around 5.

In [16], the authors proposed a call based churn-prediction technique that exhibited satisfactory predictive effectiveness when more recent call details (such as call pattern changes and contractual information) were employed for the churn prediction model construction. That technique was able to provide the ten decile lift of up to 5.4, within a one-month interval between model construction and churn prediction.

The system presented in [17] provides the ten decile lift of up to 10, using customer demographics, billing information, contract status, call detail records and service change logs. This additional information provided valuable insight in customer behavior that is reflected in higher ten decile lift values. On the other hand, it is important to notice that only a small portion of population was analyzed (around 160,000 customers), with very small churn rate (0.71%). The authors remarked that the data size was not sufficient to build a good predictive model by each customer segment. Still, this work provides useful guidelines for future improvements of churn prediction models.

A three-phase customer churn prediction technique was presented in [18]. The first phase included a supervised feature selection procedure, while the second implied the Knowledge Based System (KBS) definition through Ripple Down Rule (RDR) learner. In the final phase, a technique for Simulated Expert (SE) is proposed to evaluate the Knowledge Acquisition in KBS. Even though there were no lift values calculated in this paper, it can be noted that RDR learner achieved accuracy as high as 95%, while true positive ratio for churners was as high as 73%. In the end, SE correctly reclassified all wrongly classified observations. It is important to emphasize that the results presented in this paper were achieved by observing only 3333 customers.

From different experiments on customer churn data, it can be seen that a classifier shows different accuracy levels for different zones of a dataset. In such situations, a correlation can easily be observed in the level of classifier’s accuracy and certainty of its prediction. Ref. [19] proposes a mechanism that can estimate the classifier’s certainty for different zones within the data. This can improve the expected classifier’s accuracy. The authors present a novel churn prediction approach based on the concept of classifier’s certainty estimation using distance factor. The dataset was grouped into different zones based on the distance factor which are then divided into two categories as data with high certainty, and data with low certainty, for predicting customers exhibiting churn and non-churn behavior.

The multi-feature clustering problem, such as one presented in this paper, can be solved a bit differently, as presented in [20]. The proposed solution is based on two-stage k-means segmentation. In the first step, the authors apply k-means with Silhouette analysis for each feature to automatically decide the proper number of clusters K and assign data into different clusters. Next, the authors convert the features of each observation into a combination of the features of its nearest cluster center in each feature. The final step requires applying k-means with Silhouette analysis again on the feature combinations in order to create the final clusters. This two-stage k-means segmentation unfortunately cannot be implemented in our use case due to different business requirements and core business specifics that vary in telco operators and mobile social applications.

In [21], the authors propose a social network-based segmentation to identify strongly connected customers with high influence on their social network. The authors used two metrics (node degree and level of connectivity—derived variable based on number of calls and node degree) and defined four clusters. By targeting offers to the community leaders, the telco partner wanted to encourage customers to build social groups using their social influence. The results pointed out that churn rate decreased due to increased client satisfaction (the exact numbers were not presented), while customer spending increased.

In [22], authors described a novel diffusion model prediction scheme applicable to a single user or a small subset of users. The model introduces elements of social science into an energy-spreading diffusion algorithm. The improvements of the diffusion model include modification of the initial user energy determination using socio-metric clique theory and modifications of the energy distribution algorithm itself by including the social status theory. The resulting model produced a top decile lift of 2.5.

In conclusion, most of the papers on churn prediction in telecommunications using call data enable ten decile lift in a range from 2 to 5. In Section 5.3, we will present the results achieved by using our proposed method. The results will demonstrate that our system provides a promising basis for further development.

## 3. Methodology

In this section, we provide a detail description of the proposed model. Section 3.1 presents the method description, while Section 3.2 presents the overview of the metrics that will be used in analysis.

Customer churn prediction is binary classification problem in which all telco operator customers are divided into two possible behaviors, churn and non-churn. Additionally, the churn behavior can be classified into the two basic categories: voluntary (customer decides to leave operator) and involuntary (telco operator decides to terminate the contract) customer churn [19]. The main focus of our research will be on external voluntary customer churn (voluntary churn which will result in customer switching telco operator), which is very important for telco operator’s point of view because it may directly affect operator’s key financial indicators.

We will present a model that is based on graph theory and social network analytics that combines directed and undirected properties of clients call patterns. The clustering model will generate the client clusters and define the influencers, i.e., the users that can motivate their friends to switch operators.

### 3.1. Method Description

**Figure 1:**
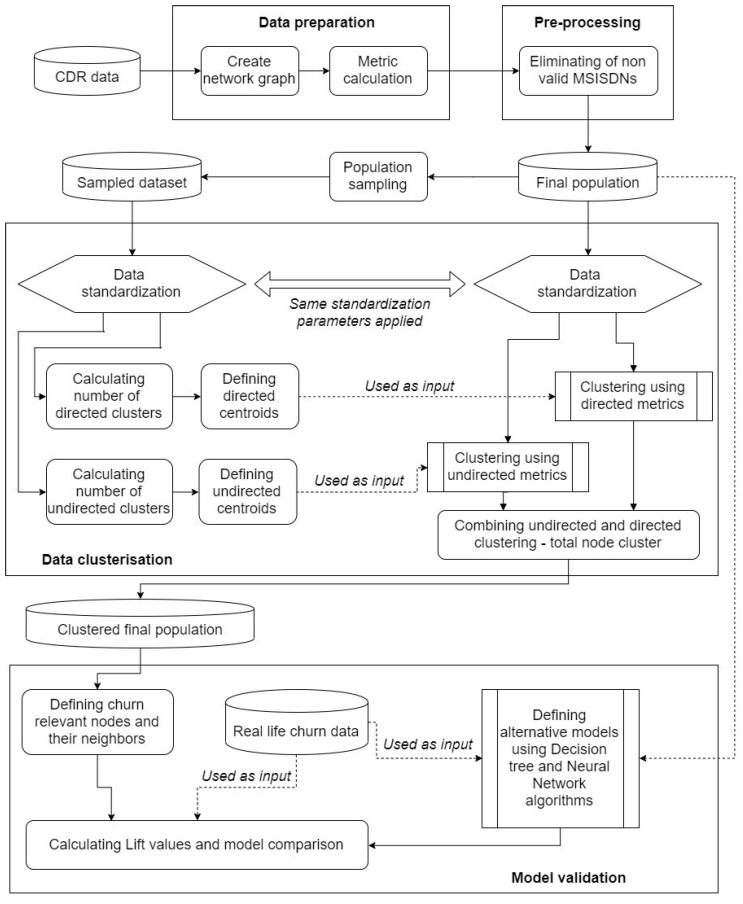
Proposed method schematic diagram.

The proposed method description is presented in this chapter. Figure 1 shows all steps necessary for creating our churn prediction model.

In the first step, data preparation is carried out; namely, we collect all required CDR data and create the network call graph. As above mentioned, each time a call is conducted on the network, descriptive information about the call is saved. This information is called a CDR. CDRs contain various information regarding specific calls, such as caller number, called number, timestamp of the interaction, duration of the call, revenue generated, etc.

We can observe each MSISDN that was recorded in a CDR as a node of the network call graph. Furthermore, we can define that edge *u*→*v* between two nodes *u* and *v* of a graph exists if our dataset contains a CDR that has those two MSISDNs as caller and called number. It is important to notice that if the analyzed dataset contains multiple CDRs in which *u* is caller and *v* is called number, we will observe all those CDRs as a single graph edge: *u*→*v*. On the other side, if we observe two CDRs where one has *u* as caller and *v* as called number, and the other has *v* as caller and *u* as called number, we will define these two CDRs as two separate edges: *u*→*v* and *v*→*u*. We will define first and second weight of each edge *u*→*v* as total number of calls between nodes *u* and *v*, and as sum of total durations of each call between nodes *u* and *v*, respectively.

Further on, we will calculate all metrics of interest that will be used for numerical representation of different call graph nodes (MSISDNs). Each node of network call graph will have the total of nine weights. Weights of the nodes are more complex to define, so they will be defined in the Section 3.2. It is important to emphasize that some of the node weights that we calculated for telco graph nodes were up to now used mostly in world-wide-web graph mining.

The second step is data pre-processing, the elimination of all non-valid MSISDNs and defining the total population final dataset that will be further processed.

As the third step, data clustering is carried out. The first task in the data clustering step is to define a sample from the final dataset. Both the sample and the final population data were standardized by using the same standardization parameters and methods. By using standardized sample data we will calculate the number of clusters that can be distinguished for both directed and undirected metrics. Ward’s minimum-variance method of clustering will be performed on sampled data and centroids will be determined for both directed and undirected metrics. Based on the determined centroids, the total population final dataset will be clustered. Finally, the combination of directed and undirected clustering results is carried out and the total node cluster is introduced.

In the fourth step—model verification, by using previously introduced total node cluster, we will precisely define the nodes that are more important regarding our churn prediction model. These nodes will be defined as Leader and Important nodes. Consequently, their neighbors will be isolated in a separate “churn” dataset. While using real-life churn data, we will also demonstrate that our model provides respectable results. Additionally, to confirm our model selection we will perform comparison with alternative modelling methods based on Neural Networks and Decision tree algorithms. It is important to notice that the alternative models will be defined by using the same data that were used for clustering model development. Finally, we will present the comparison between our clustering model and alternative models that focus on churn prediction by using ten decile lift metric.

### 3.2. Node Weights and Initial Graph Analysis

As mentioned in the introduction, to precisely define the value of a specific node, we will use nine different metrics. If we observe two nodes—*u* and *v*—let us define link weight $w_{uv}$ for link *u*→*v*. Then the link weight matrix can be defined as:

(1) $$W=\left[\begin{array}{cccc} w_{11} & w_{21} & \cdots & w_{N1}\\ w_{12} & w_{22} & \cdots & w_{N2}\\ \vdots & \vdots & \ddots & \vdots \\ w_{1N} & w_{2N} & \cdots & w_{NN} \end{array}\right]$$

where *N* represents the total number of nodes in the graph. If the nodes *u* and *v* are not connected, then link weight $w_{uv}$ is equal 0. It is important to underline that for undirected graphs, by definition: $w_{uv}=w_{vu}$. Furthermore, let $N_{u}$ represent the li node *u*. Then, nine graph metrics are [23]:

1. Node degree is defined as the total number of edges that are incident to a specific node. Node degree can be calculated both for directed and undirected graphs. For undirected graphs, node degree would be equal to the number of nodes that are connected to a specific node On the other hand, for directed graphs node degree would be equal to the sum of node in-degree and node out-degree.
2. Node in-degree in directed graphs is defined as the total number of in-edges incident to specific node.
3. Node out-degree in directed graphs is defined as the total number of out-edges incident to specific node.
4. Node first-order influence is a generalization of node degree metric that considers link weights of adjacent nodes. The general formula for first-order influence is:
  (2) $$I_{1}\left(u\right)=\frac{\sum_{v\in N_{u}}w_{uv}}{N}$$
  Node first-order influence can be calculated both for directed and undirected graphs. The main difference between these calculations is in definition of adjacent nodes, for directed graphs the neighbors are out-links.
5. Node second-order influence is a generalization of node degree metric that considers link weights of nodes that are adjacent to adjacent nodes. The general formula for second-order influence is:
  (3) $$I_{2}\left(u\right)=\sum_{v\in N_{u}}I_{1}\left(v\right)$$
  Node second-order influence can be calculated both for directed and undirected graphs, similarly like in first-order influence case.
6. Node eigenvector value is an extension of degree values in which centrality values are awarded for each node. Since not all nodes are equally important, a connection to a more important node should contribute more to centrality score than a connection to a less important node. The general formula for eigenvector value is:
  (4) $$E\left(u\right)=\frac{1}{\lambda }\sum_{v\in N}w_{uv}E\left(v\right)$$
  where λ is a constant and *E(v)* is node eigenvector value of node *v*. In matrix form, previous formula can be written as *WE = λE*, where *W* is link weight matrix. Node eigenvector value can be calculated only for undirected graphs.
7. Node authority value and node hub value represent measures of node importance. These values were introduced by Jon Kleinberg in his analysis of web pages importance and ranking [24]. This method can be used in telecommunications as well. We can say that more influential telco users are those who have a lot of incoming (or outgoing) calls (thus having larger node authority (or hub) value). The general formulas for authority and hub values are:
  (5) $$A\left(u\right)=\alpha \sum_{v\in N}w_{uv}H\left(v\right)$$
  (6) $$H\left(u\right)=\beta \sum_{v\in N}w_{vu}A\left(v\right)$$
  where α and β are constants and *A(v)* and *H(v)* are authority and hub values of node *v*, respectively. In matrix form previous formulas can be written as $WW^{T}A=\lambda A$ and $WW^{T}H=\lambda H$, where λ=αβ is a constant. Node authority value and node hub value can be calculated both for directed and undirected graphs.
8. Node in a graph is an articulation point if its removal would cause an increase in the number of connected components. Each node in the large call graph will be awarded a value 1 if it is an articulation point, otherwise it will be awarded value 0. Node articulation point value can be calculated only for undirected graphs.

Bearing in mind that some variables can be measured for undirected and other for directed graphs, we can classify nine graph metrics into three distinct groups:

1. Measures for directed graphs: Node in-degree, node out-degree, node first-order influence, node second-order influence, node authority value and node hub value. These measures are most important in telco graphs since it is important to distinguish originating and terminating number in a call.
2. Measures for undirected graphs: Node degree and node eigenvector value. These measures are not as important as measures for directed graphs in telco graphs, but still carry some valuable information regarding call graph structure.
3. Articulation point measure: Articulation point measure will be observed as separate measure because it represents a measure of graph structure and connectivity, but it does not mark a telco user as important by default. This will be explained in more detail in following example.

**Figure 2:**
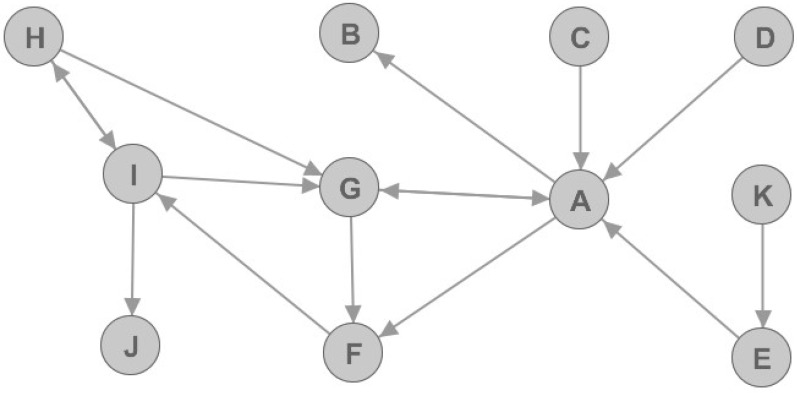
Example of simple directed graph.

Let us observe a simple directed graph that is presented in Figure 2. In Figure 2, we can see that some nodes are better connected to the rest of the graph, hence they are more important for graph structure than the others. For example, nodes A and G are more important than the others because omitting them in the graph would result in considerably decreased number of edges. Furthermore, the number of connected components in the graph would increase. The results for nine graph metrics are displayed in Table 1, and they can be used to determine the node value. It is important to notice that constants needed for metrics calculation are chosen so that the resulting values are normalized in the range between 0 and 1. Furthermore, for simplicity purposes, the weights of each edge in the graph are set to 1.

If we observe the resulting metrics we can notice that node E is marked as articulation point. However, if we observe Figure 2 we can see that node E is not very important or vital for network graph. Furthermore, we can notice that node G that is very well connected and very important, but yet it is not marked as articulation point. Thus, articulation point flag should be used for analyzing telecommunication networks, but with great caution.

Let us again check the resulting metrics. It can be noted that nodes A and G are very important: this is shown by both undirected and directed metrics. However, if we observe node B we can see that it has relatively high authority value. This is explained by the phenomenon of transferred authority. This means that if an unimportant node with no network value regarding churn prediction model in telco networks (in this case, node B) is connected to a very important hub node (in this case, node A), it will have a large authority value. Still, that node will not be an important node regarding churn prediction. Similarly, we can define phenomenon of transferred hub and transferred eigenvector values. These phenomena will be vital for later data analysis.

## 4. Data Clustering Process

### 4.1. Datasets and Data Cleansing

We analyze CDR data of a real telco operator that are collected during one month (from 1–31 March 2016). Only CDRs regarding voice calls are used for constructing the network call graph. It is important to underline that, due to the confidentiality restrictions, the data have been deterministically anonymized by encoding subscriber and recipient MSISDNs (i.e., the MSISDNs are each time coded the same). As mentioned in [10,11], very short duration calls (up to 5 s) that happened only once between two numbers in the period observed have been ignored as missed or wrong calls, since they may yield incorrect results. The CDR data contained 8.2 million nodes and 67.2 million edges and average of 8.2 edges per node. The following information was extracted while analyzing the resulting large call graph:

- Network call graph contained 948 undirected connected components;
- Network call graph contained 2.8 million directed connected components;
- Network call graph has maximum clique size of 22.

Since our objective is to create a churn prediction model for real customers, it is important to eliminate all other non-valid MSISDNs that generate a lot of traffic. Such not valid MSISDNs are emergency numbers (police, fire department, ambulance…), call centers, telemarketing centers, etc. Some of them can be recognized by using special databases that contain all operator known special numbers, but others should be found by analyzing the data. It is expected that call centers should have extremely high node in-degree and very low node out-degree. Furthermore, telemarketing centers should have extremely high node out-degree and very low node in-degree. We verified these assumptions by analyzing the data. In the first step, we calculated the degree metrics for all nodes, including already known call centers and telemarketing centers. In the second step, we grouped all known call centers and analyzed the pattern of their calls. Our goal was to set the limits for node degree metrics so that at least 90% of all known call centers were grouped together. These conditions were met for nodes that have degree above 1000 and where node in-degree is at least 100 times larger than node out-degree. When we defined such a pattern, we labelled all other nodes with similar call patterns (that currently were not labelled as not valid) as call centers as well.

A similar principle was used for telemarketing centers. In this case the proposed limits were node degree of at least 1000 and node out-degree that is at least 100 times larger than node in-degree. According to these criterions, the total of 800 nodes was marked as not valid MSISDNs.

It is important to emphasize that, while the specific values for node degree limits cannot be used as a common value in different markets (due to local market specificities, telco operator market size, etc.), the principle itself can be easily implemented in any other case.

Another possible problem presented calls where GSM gateways were used. In these cases, CDRs did not show real terminating or originating numbers. This was the case with another 200 invalid MSISDNs.

After completing pre-processing and eliminating all inadequate MSISDNs, we can create the final network call graph. That call graph contained 8.2 million nodes and 66.4 million edges and average of 8.1 edges per node. The final call graph analysis revealed the following statistics:

- Network call graph contained 1003 undirected connected components;
- Network call graph contained 2.8 million directed connected components;
- Network call graph has maximum clique size of 22.

If we closely observe the undirected connected components, it is important to notice that the largest connected component contains 99.96% of all nodes in the graph. All other connected components are much smaller, but not less important regarding churn prediction. This will be explained in the following sections.

As previously described, all nine graph metrics were calculated for the final call graph. This was performed using the SAS Enterprise Guide software for big data statistical analysis. Each MSISDN in the call graph was identified as monitored telco operator user, or as other network user. It is somewhat expected that the call patterns of Prepaid and Postpaid mobile users are not the same in volume (due to the different tariff plans and significantly different call rates). Hence separate analysis for Mobile Postpaid and Mobile Prepaid customers’ is preferred. Consequently, in the following section we will present only the detail process of Mobile Postpaid customers’ clustering using graph theory, while Mobile Prepaid users will be excluded. In total we have 1.7 million Mobile Postpaid users that will be analyzed. Finally, we expect to confirm our theory by verifying computed results using real-life churn data.

### 4.2. MSISDN Clustering

#### 4.2.1. Data Sampling and Standardization

Before we begin with node clustering we need to carry out the pre-processing of the results. This is necessary because many metrics are severely skewed, and using logarithmic and square root transformations will help achieving maximization of metrics normality.

The next step would be to choose a sample from the entire population that can be used to develop the clustering rules. Afterwards, the remainder of the population can be scored according to the sample rules. The sample was selected using simple random sampling without replacement and 170,000 nodes were chosen (10% of population).

The sample dataset was then standardized applying range standardization by formula:

(7) $$S=\frac{V-V_{min}}{V_{max}-V_{min}}$$

where *V* is a variable that is standardized, $V_{min}$ is minimum value of *V* in sample dataset, $V_{max}$ is maximum value of *V* in sample dataset and *S* is the standardized value.

#### 4.2.2. Clustering

As mentioned before, all nine graph metrics were separated in three distinct groups: measures for directed graphs, measures for undirected graphs and articulation point measure. This is the main reason for performing two separate clustering procedures, one for directed and another one for undirected metrics. Many clustering methods as a precondition require the expected number of clusters, so the further step would be to determine the number of clusters to be formed. The number of approaches for resolving this issue underscores its importance. In our work we used pseudo-T2 statistics (PST2) (introduced by Duda and Hart [25]), Cubic Clustering Criterion (CCC) (introduced by Sarle [26]) and Pseudo F statistics (PSF) (introduced by Calinski and Harabasz [27]). It should be noted that there exist no completely satisfactory methods for determining the number of clusters present, for any type of cluster analysis [28,29,30]. It is possible that the above mentioned statistics will not return the same recommendation as to the number of clusters. In that case it is necessary to look for consensus among the results.

Sarle’s Cubic Clustering Criterion (CCC) approximates the R-squared (RSQ) distribution by adopting the simplifying assumption that the clusters are hyper-cubes in a hyper-box [26]. Although this assumption is false in most cases, it still can be used to assess the null hypothesis that the data has been sampled from a uniform distribution. CCC values exceeding 2 favor rejecting the null hypothesis of no cluster structure.

The Pseudo F statistic (PSF) measures the separation among the clusters at the current level in the hierarchy. If *n* observations form *g* clusters, and *B* is the between-group sum of the squared distances between the cluster means and the grand mean vector, the PSF statistic is given by [27]:

(8) $$S=\frac{B/(g-1)}{Q/(n-g)}$$

where *Q* is the pooled within-cluster sum of squared distances between the observations and their cluster centroid.

The PST2 measures the separation between the clusters most recently joined. Any increase in the PST2 value indicates that the cluster means are increasingly different from each other and should not be joined. The number of clusters just prior to fusions producing an increase is, therefore, a potential solution. The PST2 value generated when clusters *k* and *l* are fused together into cluster *m* is given by the following equation [25]:

(9) $$PST2=\frac{W_{m}-W_{k}-W_{l}}{\left(W_{k}+W_{l}\right)/\left(n_{k}+n_{l}-2\right)}$$

where $W_{h}$ is the cluster *h* pooled within-cluster sum of squares, and $n_{h}$ is the number of members of the cluster *h*.

All of these statistical methods for calculating optimal number of clusters were implemented by using SAS Enterprise Guide software for both undirected and directed metrics. All criteria supported a three cluster solution for undirected and a four cluster solution for directed metrics.

Now, when the number of clusters is known, we will use Ward’s minimum-variance method of clustering [31] to definitely cluster the sample data. There exist alternative clustering methods, but since our idea was to minimize the variance within each cluster we have chosen to use Ward’s method. Ward’s method joins the clusters so that, at each generation, the within-cluster sum of squares is minimized over all partitions obtainable by merging two clusters from the previous generation. It is important to underline that Ward’s method is sensitive to outliers [32]. The pre-processing steps that were presented in Section 4.1 have resolved the outlier issue, hence there are no obstacles for using Ward’s method.

In the end, centroids are created for each cluster, so that later on the entire population can be easily grouped into clusters. The results of data clustering on sample level and on the entire population are displayed in Table 2.

Table 2 shows that the sample data was correctly selected since the distribution of the nodes between clusters in the sample and in the entire population is approximately the same.

**Figure 3:**
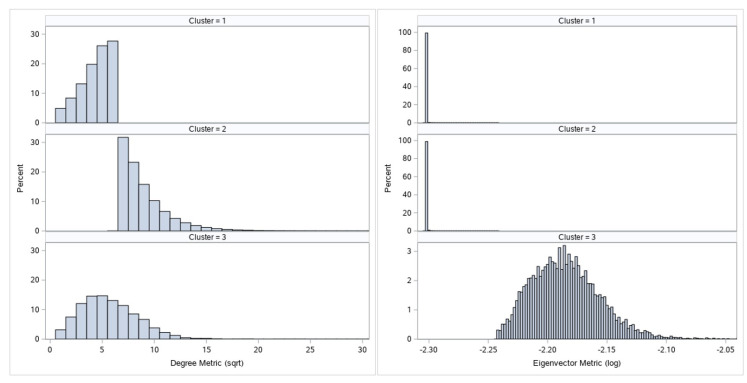
Comparative histogram plots for undirected metrics clustering.

**Figure 4:**
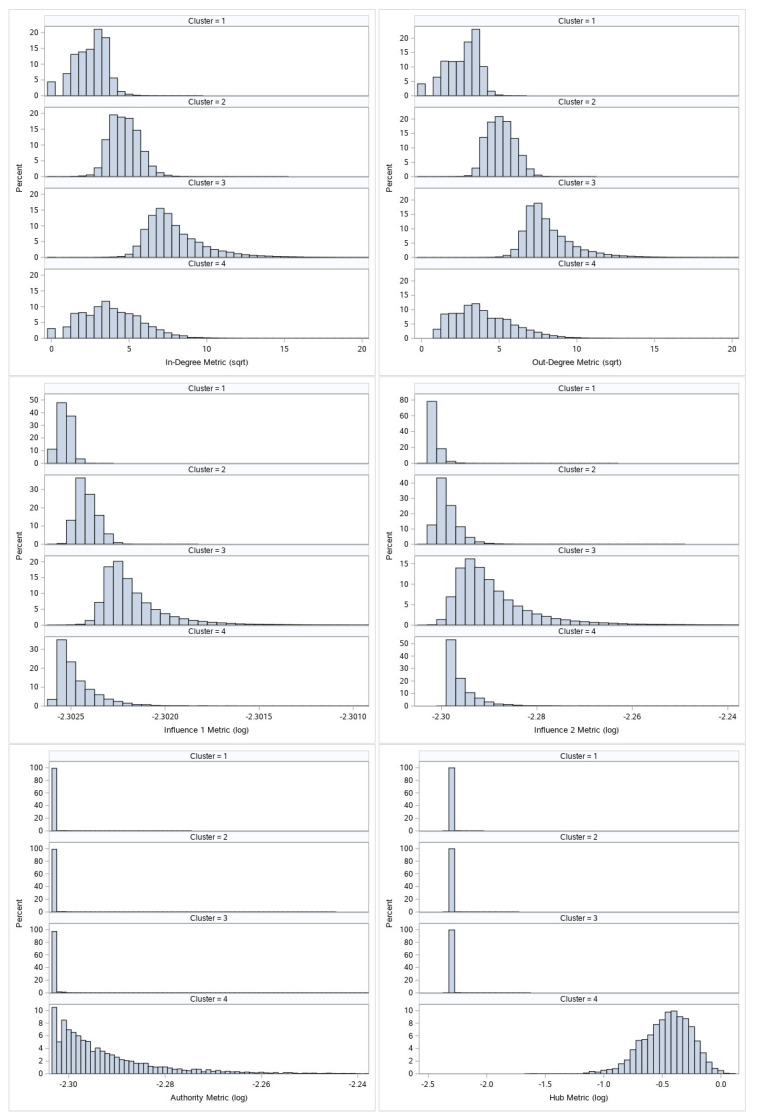
Comparative histogram plots for directed metrics clustering.

Figure 3 and Figure 4 display the comparative histogram plots for undirected and directed metrics by assigned cluster.

Each cluster is defined by its metrics characteristics and given a numeric mark. These numeric marks range from 1 to 3 for undirected graph metrics, and from 1 to 4 for directed graph metrics clustering. If we observe Figure 3, we can see that cluster 2 has larger node degree metrics than cluster 1. Furthermore, since clusters 1 and 2 have similar eigenvector values, we can say that nodes from cluster 2 are more important than nodes from cluster 1. Let us observe nodes that belong to cluster 3: they are specific only because of their eigenvector value. High eigenvector values for these nodes imply that these are well connected nodes; these nodes most likely form core of the graph. On the other hand, as mentioned before, due to the phenomenon of transferred eigenvector values not all nodes with high eigenvector value are important for our analysis. This is the reason for the undirected metrics cluster 3 s level separation:

- Nodes which by their other characteristics (node degree value) belong to cluster 1 will be downgraded to cluster 1.
- All other nodes will remain at cluster 3 and will represent the real network core and most important nodes of the network call graph by undirected metrics criteria.

Directed metrics clustering creates 4 clusters. Similarly as undirected metrics clustering, we can easily see the differences between first three clusters, and we again have to separate the real network core from the top cluster (cluster 4). Second level separation for directed metrics cluster 4:

- Nodes which by their other characteristics (in-degree, out-degree, first-order influence and second-order influence value) belong to cluster 1 or cluster 2 will be downgraded to cluster 1 or cluster 2, respectively.
- All other nodes will remain at cluster 4 and will represent the real network core and the most important nodes of the network call graph by directed metrics criteria.

We can see that nodes belonging to clusters with higher numeric marks are more important than those belonging to clusters with lower numeric marks. It is worthwhile noticing that articulation point metrics can also separate all nodes into two distinct clusters; one cluster contains nodes that are articulation points, and the other contains nodes that are not articulation points. Since our goal is to create unique clustering, we will introduce a formula that will unify all metrics:

(10) V=2D+U+A/2

where *V* represents the numeric value of the total node cluster, *D* represents directed metrics clustering numeric mark, *U* represents undirected metrics clustering numeric mark and *A* represents articulation point value. The coefficients in the previous formula were chosen so they can demonstrate the importance of directed metrics comparing to undirected metrics and articulation point value in telco operator markets. The telco networks (due to their business specifics) are directed in nature, hence the directed metrics are more important while determining client behavior (which is reflected through formula coefficients—higher coefficients for directed metrics in comparison to undirected metrics).

#### 4.2.3. Execution Times

The execution of the proposed approach is computationally demanding due to considerably large amount of data and complex operations; however, since the software used exploits parallel data processing, execution times are reasonably short. The system configuration that was used for execution consisted of 8-core 2.2GHz CPU and 32 GB RAM memory.

Nine graph metrics calculations were carried out in less than an hour. The approximate processing times were longest for node authority, hub, articulation and eigenvector metrics (around ten minutes per metric); all other metrics were calculated within minutes. Clustering process is rather demanding and hence longer run-time was needed. The most demanding task was to calculate the number of clusters in the sample data and this process took around one hour of the processing time. Afterwards, the clustering of the entire data using the respective number of clusters was performed in about half an hour.

## 5. Empirical Findings

### 5.1. Data Analysis and Prediction Models

In the previous section, we described how to create the unique clustering of nodes in a large call graph by combining directed, undirected and articulation metrics. When this clustering was applied to the entire population, we obtained the distribution of nodes as displayed in Table 3.

If we observe the number of members for each calculated value, it can be seen that there are four unique clusters regarding the whole population of nodes. The first cluster represents Followers, users that are not vital from our graph point-of-view analysis. Nevertheless, Follower nodes show some important characteristics that will distinguish them from typical nodes. The second cluster represents Standard users that are better connected to the rest of the network then Followers users, but they still are not vital for our graph. Standard users describe the behavior of a typical network user regarding our proposed churn prediction model, as it will be demonstrated in the following section. The third and the most important cluster for the proposed churn prediction model represents Leaders. These are our target nodes that we will monitor while verifying our churn prediction model. The last cluster represents Core nodes of the network call graph.

**Figure 5:**
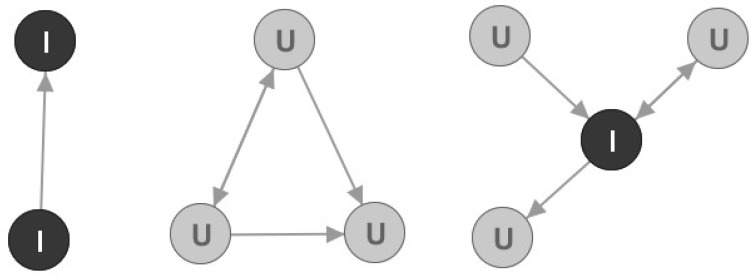
Three examples of smaller connected components.

Earlier in the paper it was mentioned that all connected components are important for our churn prediction model. However, the problem is that smaller connected components are going to have small metrics values and as such will be classified as Followers. To solve this problem, an additional analysis is needed. Let us observe Figure 5 and several examples of smaller connected components.

Nodes marked with I are marked as Important nodes, and nodes marked as U are marked as Unimportant nodes regarding our churn prediction model. Important nodes will be the focus of our analysis, just like the Leader nodes. Three analytical rules were used for this node marking:

- In connected components size 2, both nodes are marked as Important;
- In other connected components, a node is marked as Important if it is an articulation point;
- All other nodes from smaller connected components are marked as Unimportant.

In our graph analysis, there are 1970 monitored telco operator postpaid users that are not members of a large connected component and all of them are marked as Followers in the global clustering process. Out of them, 645 are marked as Important nodes.

As we mentioned before, the focus of our research is a claim that if a member from a specific group of nodes leaves our operator, then the adjacent nodes that were communicating with it would be more likely to churn. By using the previously demonstrated methods we have defined that specific group, Leaders and Important nodes.

### 5.2. Verification of the Model

The model proposed in the previous section was verified using operator disconnected numbers dataset that was observed during the period of four months (from 1 April 2016 to 31 July 2016). The disconnected numbers dataset provided us with information regarding disconnection reason, disconnection date, and, in case of number portability, the new operator that the monitored telco operator former user switched to. The process of verification was performed in the way as follows:

- Disconnection reasons for all monitored telco operator postpaid users were examined and some reasons were ignored and not treated as external churn. For example, if a customer switches from monitored telco operator Postpaid to monitored telco operator Prepaid, then from the company’s point of view it did not perform any external churn.
- Each disconnected node was analyzed and we calculated the number of its adjacent nodes that were deactivated in the forthcoming period.

The results of the data verification are presented in Table 4.

The following conclusions can be made:

1. It can be noted that churn statistics (percentage of deactivated users) of nodes from the total population as well as Standard and Leader nodes are very similar. For the reason of data confidentiality, we can show only relations between different clusters.
2. The percentage of deactivated users is smaller in Core nodes. Core nodes are tightly connected to their adjacent nodes, and as such are more difficult to be “taken” from our operator. On the other hand, Followers are loosely connected to the rest of the network and are more prone to leave our operator.
3. The percentage of deactivated users that have deactivated adjacent nodes is highest for Leader nodes. This means that if any Leader leaves our network, there is a 75% probability that some adjacent node will leave our network in the forthcoming period. This percentage is much smaller for Standard and Follower nodes.
4. Next important statistics is the average percentage of deactivated adjacent nodes per one deactivated node. This is the measure of how much one deactivated node actually degrades its surroundings. Again we can see that Leader nodes are most prone to degrade the graph structure. It should be noted that Core network nodes do not tend to degrade the graph structure. This is the case because Core nodes and their neighbors are in most cases connected to other Core nodes and as such are less probable to churn.
5. The last verification statistics displayed was the number of deactivated adjacent nodes per one deactivated node. For the reason of data confidentiality, we can show only relations between different clusters. Again, we can see that Leaders cause great degradation, and that Followers cause the smallest disruption of the graph.
6. Important nodes should be observed separately as they relate to a very small part of the population. We can see that they account for a large percentage of deactivated users, and that every deactivated user will destroy its small connected component thus confirming our theory.

It should be noted that if we analyze a case where the MSISDN number that represents a node is ported to another operator, we can see that its neighbors are also ported to that same operator in the forthcoming period.

Since decision making ultimately requires a “churn” (i.e., high probability of churn) or “no churn” (i.e., small probability of churn) prediction, we will define two distinct sets of nodes (i.e., customers). The first set will contain all nodes that are connected to Leader or Important nodes that have recently churned—they will be marked as “churn”. The second set of nodes will contain all other nodes—they will be marked as “no churn”.

### 5.3. Clustering Results

The nature of the churn prediction problem dictates a specific non-standard performance measure, since the traditional method of assessing classification accuracy of model cannot be used (e.g., if churn rate is 1% we can achieve system accuracy as high as 99% by classifying all nodes as “no churn”; however, this result would not be meaningful).

Our goal is to identify customers who were most likely to churn, so that appropriate actions could be taken to retain them (bearing in mind that only small fraction of subscribers can be contacted). In the literature regarding telecommunications industry [11,12,15,16,17] top decile lift metrics was used as the metrics of choice to compare performance of different models. The top decile lift is equal to the ratio of churners among the top ten percent of customers in terms of the likelihood score divided by the ratio of churners in the whole population. For example, suppose there are 1000 customers and the churn ratio is 4%. If the top decile lift is two, the model can capture eight churners by selecting 100 customers whose likelihood scores for churn are among top 10% of the population. In contrast, by random targeting the expected number of churners that can be captured among 10% of all customers is only 4. Therefore, the higher top decile lift, the better the model.

As we mentioned in the previous chapter, two distinct sets of customers were formed: “churn” and “no churn”. The lift statistics for our “churn” dataset are presented in Table 5. Since the total percentage of users in “churn” dataset is less than ten (because number of observed Leader or Important customers that have recently churned is not very large), we will have to expand it. “Churn” dataset will be expanded with all customers that interacted with recently churned Follower customers and one part of customers that interacted with recently churned Standard customers, which currently are not present in the “churn” dataset. The expanded dataset lift statistics are presented in Table 5. We can see that the observed lift values gradually decline. This behavior is expected because we are expanding our top performing “Churn” dataset with other customers, hence decreasing the predictive power.

It is also important to notice that the system we propose is reactive, hence it requires one initial customer to churn so it can propose other potential churners. Due to this fact, we are limited with our predictive power (we cannot predict initial customer churn). Bearing in mind this limitation, we can confirm that achieved performance is very promising. In fact, if we observe the system performance for customers marked as “Churn”, we can see that we gain very high lift values, approximately 3.9. This means that by observing only 4.5% of our total customers we have located almost 20% of all churners. As expected, the best lift values are achieved while observing customers that interact with recently churned Important nodes, up to 45.8. This means that one small subset of customers is very vulnerable and likely to churn, so advanced retention program is required.

## 6. Discussion

To further emphasize the selected method, we will create comparative models using the same data. Precisely, in order to confirm the choice of clustering for the data modelling, we will conduct comparative analysis using two additional methods. The choice of additional methods was made based on statistical methods that were used in the related work; hence, neural network and decision tree modelling were selected. It is important to underline that the both models were used on the same dataset that was previously used in the clustering process, with one small addition: we created Deactivation flag with values 1 and 0 (client performed/did not perform churn in the period observed). This flag is the target variable that new models are going to predict. Additionally, a significant number of different model configurations for each respective model technique were tested and the ones that provide the best results, respecting the compilation time constraints (the compilation time was supposed to be similar to the clustering one), were finally used.

Both model techniques were implemented using the similar principle: in the first step we created an oversampled sample from the final dataset. The oversampled dataset contained 33% of observations with the flag equal to 1 and 67% of observations with the flag equal to 0. This oversampled dataset will be used for the model creation and validation; on the other hand, the model testing will be performed on the whole dataset. In the second step, we created the training (70% of observations) and validation (30% of observations) datasets using the oversampled data. The training and validation datasets are required for the model generation; the testing dataset will be used for the model ranking purposes.

As previously mentioned, bearing in mind the type of our target variable, multiple different configurations for creating the decision tree model can be used. For example, there are multiple algorithms for decision tree splitting criterion, such as criterion based on Gini index [33], chi-squared test [34], entropy [35], etc. We used the two algorithms based on the Gini index and the chi-squared test. Hence, based on splitting criteria, Classification and Regression Trees [33] (CART) and Chi-square Automatic Interaction Detector [34] (CHAID) algorithms for decision tree learning were used. We also analyzed the impact of changing the time of Bonferroni adjustment to the moment before or after the splitting variable was chosen [34]. Additionally, different values for the maximum number of branches and the maximum tree depth were used. When the final models were generated, the top decile lift metric of testing data was calculated in order to compare the results with our clustering model.

Multiple neural network models were also generated. We analyzed various network architecture types, such as:

- Linear Perceptron (LP);
- Multilayer Perceptron (MLP);
- Radial Basis Function (RBF) Networks.

In our work we analyzed both Ordinary RBF (ORBF) and Normalized RBF (NRBF) Networks [36]. Additionally, we analyzed the effect of different combination functions used for constructing radial basis function network, such as functions with:

- Unequal Width and Height (UN);
- Unequal Widths (UW);
- Equal Width and Height (EQ);
- Equal Widths (EW);
- Equal Height (EH);
- Equal Volumes (EV).

Furthermore, we analyzed the impact of various numbers of hidden units (units in hidden layer). Using each of the defined models, the top decile lift metric of testing data was calculated. The top decile lift metric was chosen as the metric for model comparison.

Both the decision tree and the neural network model were implemented in SAS Enterprise Miner software. The final lift values are presented in Table 6 and Table 7.

The final results demonstrate that the highest top decile lift value for decision tree type models (2.49) can be achieved with CHAID algorithm with 73 nodes, a maximum of four branches, and a depth of six, while Bonferroni adjustment was performed after the split was chosen. On the other hand, neural network models have slightly better performance regarding ten decile lift in comparison to decision tree models. The best neural network configuration is the Normalized Radial Basis Function Networks—Equal Height (NRBFEH) with six neurons in hidden layer. Yet again, the achieved lift value is 2.65 and that is still lower than our proposed clustering based model. The results confirm our main thesis that the most suitable model for the collected data is based on the clustering of customers.

## 7. Conclusions

Social network analytics using graph theory can be used extensively in a wide range of applications and disciplines, such as business intelligence [37], marketing [38], healthcare [39], fraud detection (automobile insurance fraud detection [40], fraud detection in payment processing [41], etc.), web communication analysis [42], link-prediction [43], telecommunications [44], churn prediction [45], banking industry [46], etc. Since telecommunication industry is highly competitive, it is vital for every telco operator to gain as much as possible the insight into their customers’ behavior. A research study [47] showed that approximately 75% of defecting customers tell their negative experiences to at least one other person. Therefore, the information about the communication partners of a customer and their decisions to churn might support the prediction of a customer’s churn likelihood. Social network analysis can ensure that in-depth understanding of every customer.

The mainstream approach to churn prediction considers each customer individually, and dozens to hundreds of complex Key Performance Indicators (KPIs) are generated per customer. These KPIs cover the customer’s personal characteristics, billing information, contract expiry, as well as the trends in their call activities over a monitored period. Other churn prediction systems monitor customers’ calls to the telco operator’s call center; they monitor their speech and try to determine the customer’s dissatisfaction level, hence estimating churn. Finally, some papers are based on social network analytics and define the groups of customers that may affect churn (for example [11], or [15]).

In our paper, we presented the churn prediction model for a telco operator that was based on the principles of social network analytics, clustering and graph theory. Firstly we generated a large network graph using one month of real voice call CDRs. We defined nine important graph metrics that we divided into three groups: undirected and directed metrics, and articulation point. In the next step, we eliminated all non-valid MSISDN numbers and performed two separate clustering processes. We introduced the formula for joining two clustering results and defined four overall clusters (Follower, Standard, Leader and Core), and one additional sub cluster (Important). We defined the group of certain influencers (Leader and Important nodes), whose neighbors are vulnerable in case the influencer performs churn. By using the real-life churn data we located a number of influencers that have performed churn in a monitored period in order to validate our claim. Our theory that a large number of their neighbors will churn in the following period as well was confirmed by using real-life churn data. In the end, we compared our model with alternative modelling techniques like decision tree modelling and neural networks.

We would like to underline that even though it is somewhat expected that homophily influences churn in telco operator network (due to the fact that friends of churners are more likely to churn themselves) we managed to quantify this effect. Additionally, we have concluded that one special client segment (clients that interact with Core network users) is not very likely to perform churn, since it is tightly connected to the network graph core. To summarize, the expected homophilic behavior is not same for clients in different segments.

It is important to emphasize that clustering based churn models, such as the one presented in this paper, can be easily combined with other churn prediction models that are based on analysis of other relevant information. For example, by combining the results of our model and some customer satisfaction based churn model (like the one presented in [47], where price, inconvenience, core system failure, etc. were analyzed), the churn prediction success rate could be increased even more.

The conclusions of this work can be used as guidelines for some other related work regarding social network analytics, but it is very important to take into account that every different application of social network analytics requires an extensive knowledge of its social and graph characteristics that are important for creating a successful prediction model.

## Figures and Tables

**Table 1:** Resulting nine metrics for example of simple directed graph.

Node	Undirected		Directed						Articulation
	Deg.	EV	Deg. In	Deg. Out	Inf. 1	Inf. 2	Hub	Auth.	
A	6	0.96	4	3	0.27	0.27	1.00	0.63	1
B	1	0.30	1	0	0.00	0.00	0.00	0.41	0
C	1	0.30	0	1	0.09	0.27	0.30	0.00	0
D	1	0.30	0	1	0.09	0.27	0.30	0.00	0
E	2	0.33	1	1	0.09	0.27	0.30	0.00	1
F	3	0.87	2	1	0.09	0.27	0.16	0.67	0
G	4	1.00	3	2	0.18	0.36	0.62	1.00	0
H	2	0.57	1	2	0.18	0.45	0.64	0.33	0
I	4	0.84	2	3	0.27	0.36	0.80	0.33	1
J	1	0.26	1	0	0.00	0.00	0.00	0.33	0
K	1	0.10	0	1	0.09	0.09	0.00	0.00	0

Deg.—Degree; EV—Eigenvector; Inf.—Influence; Auth.—Authority.

**Table 2:** Distribution of MSISDNs by cluster in undirected and directed metrics clustering.

**Cluster**	**Undirected**	
	**Percentage of Members in Sample**	**Percentage of Members in Population**
1	53.48	56.89
2	46.13	42.72
3	0.39	0.39
**Cluster**	**Directed**	
	**Percentage of Members in Sample**	**Percentage of Members in Population**
1	42.53	39.90
2	41.04	45.25
3	16.07	14.47
4	0.36	0.38

**Table 3:** Total node distribution between final clusters.

Directed	Undirected	Articulation	Percentage of Members	Value	Unique Cluster Name
1	1	0	28.0125	3	FOLLOWER
1	1	1	12.0505	3.5	
1	2	0	0.0094	4	
1	2	1	0.0111	4.5	
2	1	0	7.9166	5	STANDARD
2	1	1	9.1662	5.5	
2	2	0	10.4609	6	
2	2	1	17.7645	6.5	
2	3	0	0.0540	7	LEADER
2	3	1	0.0230	7.5	
3	2	0	3.4634	8	
3	2	1	11.0023	8.5	
3	3	0	0.0023	9	CORE
3	3	1	0.0040	9.5	
4	2	0	0.0003	10	
4	2	1	0.0012	10.5	
4	3	0	0.0314	11	
4	3	1	0.0265	11.5	

**Table 4:** Data verification results for unique clusters.

Unique Cluster Name	Total % of Users	% of Deac. Users	% of Deac. Users that Have Deac. Adj. Nodes	Average % of Deac. Adj. Nodes per Deac. Node	Number of Deac. Adj. Nodes per Deac. Node
FOLLOWER	40.05	1.27 B	31.3	25.7	A
STANDARD	45.31	0.8 B	48.9	30.4	4.09 A
LEADER	14.54	0.88 B	74.7	38.2	16.35 A
CORE	0.07	0.32 B	57.1	2.6	1.01 A
IMPORTANT	0.03	3.38 B	88.2	100	-
Total population	100	B	43.2	33.0	4.08 A

%—Percentage; Deac.—Deactivated; Adj.—Adjacent.

**Table 5:** Lift statistics; “Churn” dataset and top decile lift.

Dataset	Total Percentage of Users	Lift
Interact with Important	0.002	45.80
Interact with Leader	4.543	3.88
“Churn”	4.545	3.89
“Churn” + Interact with Follower	6.815	3.67
“Churn” + Interact with Follower and Standard	10	2.80

**Table 6:** Decision tree models; top decile lift.

Decision Tree Type	Tree Assesment Method	Max. Depth	Max. Branch	Number of Nodes	Lift
SAS Default Tree	SAS Default	6	2	65	2.38
SAS Default Tree	Average Square Error	6	2	65	2.42
CHART	Average Square Error	10	2	317	2.46
CHART	Average Square Error	6	2	101	2.48
CHAID	SAS Default	6	6	83	2.48
CHAID	Average Square Error	6	4	73	2.49

**Table 7:** Neural network models; top decile lift.

Neural Network Type	Combinational Functions	Lift
LP	N/A	2.47
MLP	N/A	2.56
ORBF	EQ	2.41
ORBF	UN	2.48
NRBF	EQ	2.57
NRBF	EV	2.51
NRBF	EH	2.65

## Abbreviations

The following abbreviations are used in this manuscript:

CART	Classification and Regression Trees
CCC	Cubic Clustering Criterion
CDR	Call Data Record
CHAID	Chi-square Automatic Interaction Detector
EH	Equal Height
EQ	Equal Width and Height
EV	Equal Volumes
EW	Equal Widths
LP	Linear Perceptron
MLP	Multilayer Perceptron
MSISDN	Mobile Station International Subscriber Directory Number
PSF	Pseudo F statistics
PST2	Pseudo-T2 Statistics
RBF	Radial Basis Function
RSQ	R-Squared
UN	Unequal Width and Height
UW	Unequal Widths
